# American International Congress on Tuberculosis

**Published:** 1906-06

**Authors:** 


### AMERICAN INTERNATIONAL CONGRESS ON TUBERCULOSIS.

The following letter has been sent to every State medical association and health organization, boards of health, etc., in the States of North and South and Central America and the Dominion of Canada. Already many favorable and flattering responses have been received and the promise of a large attendance and many important papers. The management feels assured of such a cordial response and co-operation as to guarantee the success of the meeting. It will mark an epoch in the crusade against consumption:

AMERICAN INTERNATIONAL CONGRESS ON TUBERCULOSIS.

Office of the Council, 100 William Street.

New York, June 1, 1906.

Esteemed Colleagues: We have the honor to inform you that we are instructed by the Committee on Invitations to advise you that your body is cordially invited to send delegates to the meeting of the American International Congress on Tuberculosis, to be held in the City of New York, November 14, 15, and 16, 1906, next, and to send a list of the same as soon as convenient to enable our committee to arrange for a reduced transportation for the same.

It is highly desirable that the efforts of sanitarians and of all enlightened humanitarians, lay and professional, should be unified and concentrated in the endeavor to limit the spread, and as far as may be possible, to remove the causes of the great scourge of the human family.

There is, and should be, no spirit of rivalry. All the organizations for this laudable work should co-operate for the accomplishment of the great end sought.

With assurances of high esteem and regard, and an earnest desire that every organization interested or engaged in this conflict with tuberculosis may combine their efforts in a common cause, we remain, Very respectfully yours,

F. E. Daniel, M. D., President,
Austin, Texas.
Matthew M. Smith, M. D., Secretary,
Austin, Texas.
Clark Bell, LL. D., Treasurer,
39 Broadway, New York.
H. Edwin Lewis, M. D.,
Vice Chairman of Council,
100 William St., New York.

[Exchanges will oblige me by copying the above.—Editor Texas Medical Journal.]

---

The Fourth of July is nearly here. Remember the deadly toy pistol and the deadly firecracker. Every philanthropic doctor should enlighten the families in his community on the subject of tetanus and warn them against it. No one knows why, but the fact is recognized that lockjaw is far more apt to result from even seemingly trifling wounds from firecrackers and toy pistols than from any other wound; and lockjaw is *uniformly fatal.* Every physician should work for the total suppression of Fourth of July celebrations by such deadly nuisances. The only possible chance to save the life of a child or other person wounded by these things is to promptly treat the wound, however small, antiseptically. Warn parents to suppress the firecracker and the toy pistol. They are more dangerous than dynamite. Prevention is possible; cure, "well, hardly ever."

